# Pharmacological Influences on the R2 Blink Reflex Response in Healthy Participants: A Systematic Review

**DOI:** 10.1111/ejn.70646

**Published:** 2026-07-30

**Authors:** Josh Murphy, Joy Krecke, Celia Morgan, Paul H. Strutton, Kirsty Bannister, Sam W. Hughes

**Affiliations:** ^1^ Department of Clinical and Biomedical Sciences University of Exeter Exeter UK; ^2^ Department of Psychology University of Exeter Exeter UK; ^3^ The Nick Davey Laboratory, Human Performance Group, Division of Surgery, Department of Surgery and Cancer, Faculty of Medicine Imperial College London London UK; ^4^ Department of Life Sciences, South Kensington Imperial College London London UK

**Keywords:** neurophysiology, nociception, noradrenaline, pain modulation, pharmacology, R2 blink reflex

## Abstract

The R2 blink reflex (BR) is a polysynaptic brainstem‐mediated response that can be activated by innocuous and noxious electrocutaneous stimulation of the supraorbital nerve and has been used as a biomarker across pharmacological studies. Whilst standard patch electrodes activate both the tactile (R1) and nociceptive (R2) components of the blink reflex, concentric electrodes preferentially evoke a nociceptive‐specific response (NBR) by isolating the R2 component. This preregistered systematic review (CRD420251014203) aims to assess the pharmacological modulation of nociceptive R2 responses as measured by both standard patch and concentric electrodes in healthy participants. Following PRISMA guidelines, systematic searches of PubMed, Cochrane and Ovid identified studies examining R2 responses in healthy adults receiving a pharmacological intervention. Data were extracted on study design, drug category, stimulation electrode, dosage and outcome measures. Nine studies met inclusion criteria, which included eight distinct drug classes. Limited effects on the R2 response were found for opioids, selective serotonin receptor agonists, nonsteroidal anti‐inflammatory drugs and ketamine. In contrast, α2‐adrenoreceptor modulators, benzodiazepines, nitrous oxide and selective adenosine A1 receptor agonists demonstrated effects. The stimulation electrode used did not affect the impact of intervention on nociceptive R2 responses where comparable studies were available. These findings indicate that nociceptive R2 components of the BR, whether evoked using standard or concentric electrodes, exhibit comparable sensitivity to pharmacological modulation. Future research should further delineate the contributions of local and descending modulatory systems to nociceptive‐specific and tactile processing within trigeminofacial circuits.

## Graphical abstract

Pharmacological modulation of the R2 blink reflex was comparable whether responses were elicited using standard patch or concentric electrodes. Across nine studies, noradrenergic and GABAergic interventions produced the most consistent effects, supporting the R2 blink reflex as a sensitive biomarker of trigeminofacial nociceptive processing.

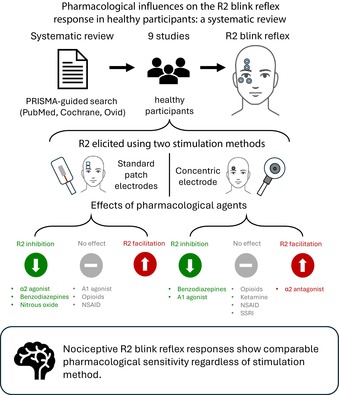

**Abbreviations**
ASA: acetylsalicylic acid
AUC: area under the curve
BR: R2 blink reflex
BZD: benzodiazepine
DPMS: descending pain modulation system
FMN: facial motor nucleus
HFS: high‐frequency stimulation
NBR: nociceptive blink reflex
NS: nociception‐specific
NSAID: nonsteroidal anti‐inflammatory drugs
OP: opioid
RF: reticular formation
SNRI: serotonin–noradrenaline reuptake inhibitor
STN: spinal trigeminal nucleus
WDR: wide dynamic range

## 1 Introduction

The R2 blink reflex (BR) is a polysynaptic brainstem reflex, which can be elicited by electrocutaneous stimulation of the supraorbital branch of the trigeminal nerve (Kofler et al. 2024). When evoked using a standard patch electrode, it comprises an early R1 component mediated by low‐threshold Aβ afferents, reflecting non‐nociceptive (i.e., tactile) processing, followed by a later bilateral R2 component that primarily reflects nociceptive processing via convergent Aβ and Aδ inputs onto wide dynamic range (WDR) neurons in the spinal trigeminal nucleus (STN) (Murphy et al. 2025). Nociception‐specific concentric electrodes can instead be used to preferentially activate Aδ fibres, which also project to nociception‐specific (NS) neurons within the STN (Ellrich 2002) evoking a nociceptive blink reflex (NBR) that bypasses the R1 component and allows more selective interrogation of the nociceptive contribution to the R2 response.

Pharmacological agents can modulate the R2 response by acting at multiple levels of the brainstem trigeminal system, which is subject to both local and descending modulatory influences (Kofler et al. 2024). The R2 component has been widely used as a neurophysiological read‐out of nociceptive processing within these pathways in pharmacological studies and has therefore been identified as a biomarker in the development of centrally acting pain therapies (Kofler et al. 2024; Leone et al. 2022). Previous attempts to pharmacologically probe the R2 response include modulation of monoaminergic and opioidergic systems, which are involved in the descending control over brainstem nociceptive processing (Tavares et al. 2021). Others have targeted local modulatory processes within the brainstem through inhibitory GABAergic and excitatory glutamatergic mechanisms (Gray 2013; Tamai et al. 1986) alongside inflammatory processes present within polysynaptic trigeminofacial circuits (Kofler et al. 2024).

Identifying which pharmacological agents most consistently modify the R2 component of the reflex will help to identify key neurotransmitter systems that influence nociceptive transmission within trigeminofacial pathways. However, it remains unclear whether the sensitivity of the R2 component to pharmacological manipulation depends on the method of stimulation, given that standard patch electrodes recruit mixed Aβ and Aδ afferents, whereas concentric electrodes preferentially activate nociceptive Aδ inputs (Kaube et al. 2000).

Accordingly, this systematic review examines the use of the R2 component of the BR pathway as an outcome measure in pharmacological studies, with direct comparison between responses elicited using standard patch and NS concentric electrodes. We aimed to characterise pharmacological modulation of the R2 component across studies targeting both descending (monoaminergic and opioidergic) and local (inhibitory and excitatory) mechanisms.

## 2 Methods

This systematic review was performed using the Preferred Reporting Items for Systematic Reviews and Meta‐Analyses (PRISMA) guidelines. The review was preregistered with PROSPERO. Deviation from the preregistered protocol was necessary as we removed the nociceptive withdrawal reflex aspect, moving the focus to brainstem mechanisms rather than methodology; a meta‐analysis was also removed as it was determined to be impossible with the current literature (PROSPERO 2025 CRD420251014203; available from https://www.crd.york.ac.uk/PROSPERO/view/CRD420251014203).

### 2.1 Search Strategy

The primary search was conducted using PubMed, Cochrane and Ovid. We used the search terms ‘[Nociceptive blink reflex OR R2 blink reflex AND healthy]’ whilst limiting to humans and pharmacologic actions in Ovid. We used the search terms ‘[((((Nociceptive blink reflex) OR (R2 blink reflex)) AND (healthy)) AND (humans)) AND (pharmacologic actions)]’ in PubMed. We used the search terms: Nociceptive blink reflex OR R2 blink reflex AND healthy AND modulation in Title Abstract Keyword OR R2 blink reflex in Title Abstract Keyword AND ‘pharmacologic action’ in Title Abstract Keyword AND Human in Title Abstract Keyword AND modulation in Title Abstract Keyword—(Word variations have been searched). Duplicates were removed.

**FIGURE 1:**
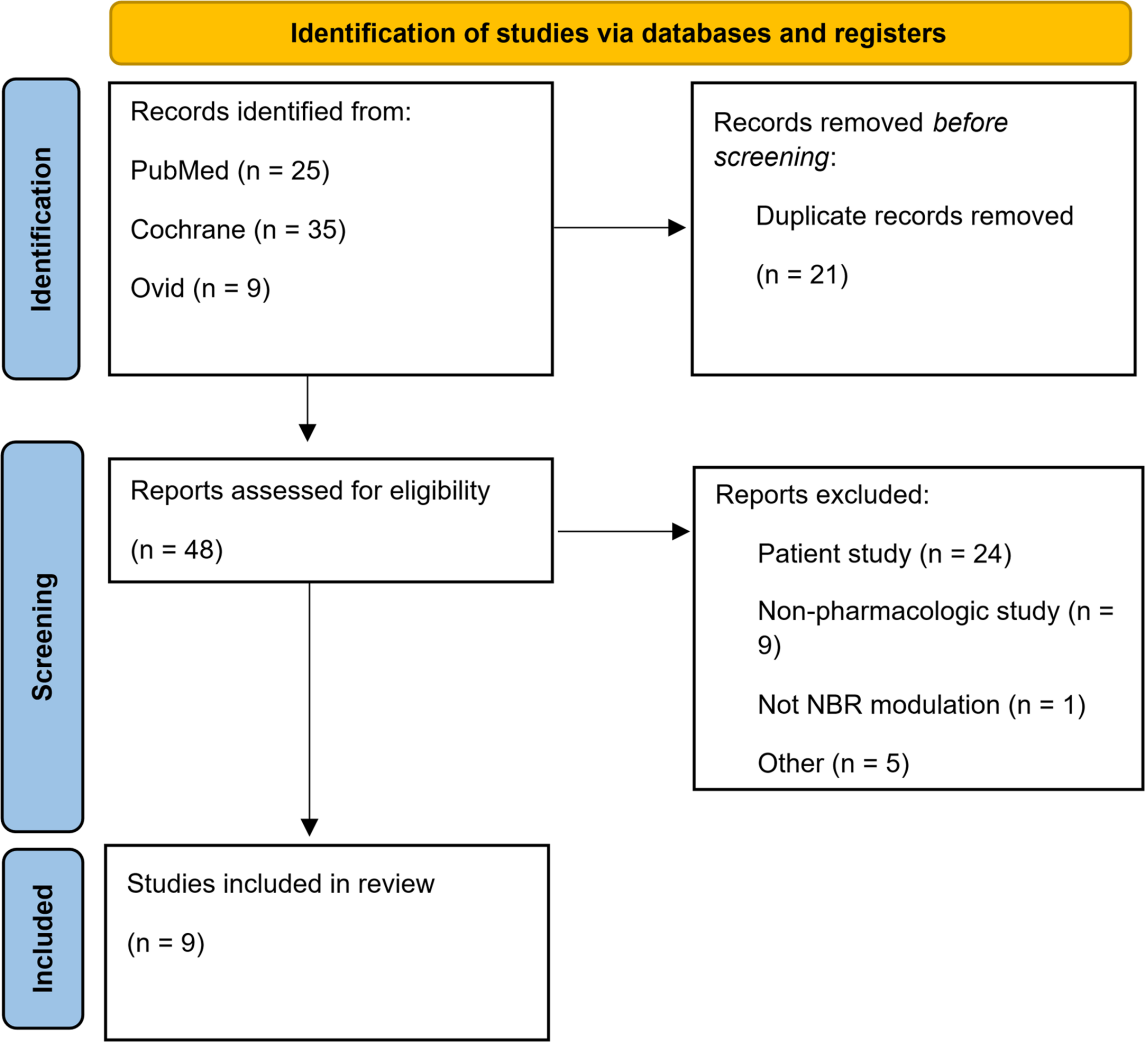
PRISMA flowchart. The initial search was performed across PubMed, Cochrane and Ovid databases. Duplicate entries were excluded (*n* = 21), and the remaining full‐text articles were assessed based on established inclusion and exclusion criteria. The reports which met these criteria (*n* = 9) were selected for data extraction and subsequent analysis.

This assessment was completed by screening each article against the specified criteria, which was completed by J.M. After this primary search, no other modified search terms or manual searches identified further studies, which met the inclusion criteria. The results of these searches and numbers included are outlined in Figure 1. Searches were limited to the English language since the most recent update of the databases on 8 April 2025.

### 2.2 Study Eligibility

The results (*n* = 48) were assessed based on predefined inclusion and exclusion criteria. Studies were included if they primarily focused on modulating the R2 component with pharmacologic agents in healthy human participants. Studies were excluded if they investigated patient cohorts to ensure the review is focused on the basic mechanisms of the BR, studied anaesthetic doses or if they were review articles.

### 2.3 Data Extraction

For all studies included in the final analysis, data extraction was conducted by J.M., capturing key methodological and outcome details. Extracted variables included sample size, intervention type, dose, analysis type, study design, conditioning stimulus used (where applicable), number of male and female participants and participant age.

The risk of bias for all identified studies was evaluated using the Cochrane risk of bias assessment tool (Higgins et al. 2011). Two authors (J.M. and J.K.) independently assessed the risk of bias for each study, examining all six domains outlined by the tool. Any discrepancies between assessments were discussed and resolved through consensus. The final risk of bias evaluation was summarised and reported for each domain.

### 2.4 Data Synthesis

A systematic narrative synthesis was conducted to evaluate the effects of different pharmacological interventions on the R2 component of the BR. Included studies were required to report at least one baseline reflex measurement followed by a post‐intervention assessment. The synthesis focused on the type and dose of the intervention, and outcomes were summarised in terms of their reported effects on the R2 response.

## 3 Results

The primary search conducted using databases provided 69 results. These results were processed by removing duplicates (*n* = 21), leaving 48 publications to be reviewed. Each publication was then assessed to determine whether it met the inclusion and exclusion criteria. After this, nine studies were identified and taken forward. Search results are outlined in Figure 1.

### 3.1 Summary of Included Studies

The R2 response was measured in healthy participants using a variety of interventions, with all but one using a placebo‐controlled design. Five studies were double‐blinded, with three studies being single‐blind on the side of the participant.

For this section, discussing pharmacological interventions trialled on the R2 component, we identified 10 different pharmacologic actions. There were two studies with opioids (OPs) as a focus, three studies using nonsteroidal anti‐inflammatory drugs, two studies using a form of benzodiazepine (BZD), one study using a dissociative anaesthetic at a nonsedative dose in line with our inclusion criteria, one study using an α2‐adrenergic receptor antagonist, one study using an α2‐adrenergic receptor agonist, one study using a selective serotonin receptor agonist, one study using a selective adenosine A1 receptor agonist and one study using analgesic gas. These studies have been divided further into stimulation electrode type used to elicit the R2 response, allowing evaluation of both standard patch and NS concentric electrodes. A further breakdown of each study can be found in Table 1, and a breakdown of study results can be found in Table 2.

**TABLE 1:** Study‐by‐study characteristics. Breakdown of each study's participant population, study design, intervention type and stimulation electrode type used.

Author	Study design	*N*	Age	Intervention	Drug type	Stimulation electrode
Vo and Drummond (2016)	Double‐blind, placebo‐controlled crossover	22 (21 final)–13 male	18–52 years	Yohimbine	α2‐adrenergic receptor antagonist	Two custom‐built concentric electrodes
Marin et al. (2015)	Single‐centre, randomised, double‐blind, placebo‐controlled, crossover, four‐arm study	31 (21 final)–21 male	21–37 years	Diazepam	Benzodiazepine	Custom built concentric electrode
				Fentanyl	Opioid	
				Ketamine	Dissociative anaesthetic	
Katsarava et al. (2004)	Double‐blind, placebo‐controlled, crossover	30 healthy participants—11 male	25.7 ± 4.8 years	Acetylsalicylic acid (ASA)	NSAID (aspirin)	Planar concentric electrode
				Oral zolmitriptan	Selective serotonin receptor agonist	
Giffin et al. (2003)	Double‐blind, randomised, placebo‐controlled, crossover	26 (12 final)–12 female	20–36 years	GR79236	Selective adenosine A1 receptor agonist	Standard electrode
						Custom‐built planar concentric electrode
Palmeri et al. (2000)	Within‐subject, controlled	10–5 male	22–50 years	Clonidine	α2‐receptor agonist	Single cathodal stimuli (avoiding activating nociceptive afferents)
Ferracuti et al. (1994)	Single‐blind, placebo‐controlled, crossover	6 male	25–38 years	Piroxicam	NSAID	Unclear—10 times sensory threshold to ensure painful sensation
				Lysine acetylsalicylate	NSAID (aspirin)	
Fabbri et al. (1992)	Single‐blind, placebo‐controlled, crossover	6–4 male	25–38 years	Piroxicam	NSAID	Unclear—10 times sensory threshold to ensure painful sensation
				Naloxone	Opioid antagonist	
Cruccu et al. (1991)	Single‐blind, placebo‐controlled, crossover	6 male	21–28 years	Fentanyl	Opioid	Unclear—10 times sensory threshold to ensure painful sensation
				Naloxone	Opioid antagonist	
				Diazepam	Benzodiazepine	
				Flumazenil	Benzodiazepine reversal	
Willer et al. (1985)	Double‐blind, randomised, crossover	7–6 male	27–41 years	Nitrous oxide	Analgesic gas	Surface electrodes (2–3 times sensory threshold for slight pain sensation)
				Naloxone	Opioid antagonist	

**TABLE 2:** Study‐by‐study summary of findings. Overview of the drugs used, the doses they were administered in and their effect on the NBR and BR, as reported by the studies.

Author	Intervention	Dose	Effect on NBR	Effect on BR
Vo and Drummond (2016)	Yohimbine	16 mg (oral)	Approximate increase 17% (interpreted from graph)	—
Marin et al. (2015)	Diazepam	0.03 mg/kg + 2 increments of 0.02 mg/kg (I.V.)	41.2% reduction	—
	Fentanyl	0.37 μg/kg + 2 increments of 0.37 μg/kg (I.V.)	No effect	—
	Ketamine	0.028 mg/kg + 2 increments of 0.028 mg/kg (I.V.)	No effect	—
Katsarava et al. (2004)	Acetylsalicylic acid (ASA)	1000 mg (I.V.)	No effect	—
	Oral zolmitriptan	5 mg (oral)	No effect	—
Giffin et al. (2003)	GR79236	10 mg/kg (I.V.)	17% reduction ipsilaterally, 20% reduction contralaterally	—
Palmeri et al. (2000)	Clonidine	0.5 μg/kg in 5‐mL saline (I.V.)	—	18% reduction
Ferracuti et al. (1994)	Piroxicam	40 mg (I.M.)	—	No effect
	Lysine acetylsalicylate	500 mg (I.V.)	—	No effect
Fabbri et al. (1992)	Piroxicam	40 mg (I.M.)	—	No effect
	Naloxone	2 mg (I.V.)	—	No effect
Cruccu et al. (1991)	Fentanyl	1.5 mg (I.M.)	—	No effect
	Naloxone	0.8 mg (I.V.)	—	No effect
	Diazepam	10 mg (I.V.)	—	60% reduction
	Flumazenil	0.5 mg (I.V.)	—	Partial reversal
Willer et al. (1985)	Nitrous oxide	101/min (33% NO, 66% oxygen)	—	83% reduction
	Naloxone	1.2 mg; 3‐mL total doses (I.V.)	—	No reversal

#### 3.1.1 Studies Using Concentric Electrodes

##### 3.1.1.1 OPs (Fentanyl)

Marin et al. (2015) analysed the effect of fentanyl on the NBR R2 response. They used a single‐centre, randomised, double‐blind, placebo‐controlled, crossover, four‐arm study where the stimulation was administered with a concentric electrode at 0.5–1.8 mA, which was 2–4 times the individual threshold for pin‐prick perception. The response was measured using area under the curve (AUC). The initial dose of fentanyl was 0.37 μg/kg with two additional increments of 0.37 μg/kg each administered over 2 min, giving a final dose of 1.11 μg/kg. They found no difference between fentanyl and placebo.

##### 3.1.1.2 BZD (Diazepam)

Marin et al. (2015) used a randomised, double‐blind, placebo‐controlled, crossover design where the stimulation was administered with a concentric electrode at 0.5–1.8 mA, which was 2–4 times the individual threshold for pin‐prick perception. The response was measured using AUC. Diazepam was administered via I.V. with an initial dose of 0.03 mg/kg with two subsequent increments of 0.02 mg/kg each. They found that diazepam significantly reduced the ipsilateral NBR R2 response.

##### 3.1.1.3 Dissociative Anaesthetic (Ketamine)

Marin et al. (2015) used a randomised, double‐blind, placebo‐controlled, crossover design where the stimulation was administered with a concentric electrode at 0.5–1.8 mA, which was 2–4 times the individual threshold for pin‐prick perception. The response was measured using AUC. I.V. Ketamine was administered with an initial dose of 0.028 mg/kg with two subsequent identical increments of 0.028 mg/kg and showed no effect on the R2 component of the NBR in their healthy sample.

##### 3.1.1.4 Nonsteroidal Anti‐Inflammatory Drugs (Acetylsalicylic Acid [ASA])

Katsarava et al. (2004) used a double‐blind, placebo‐controlled crossover design where the R2 NBR was elicited using an intensity 1.5 times the individuals pain threshold using a concentric electrode and analysed using AUC. ASA was administered via I.V. at a dose of 1000 mg. ASA showed no difference to placebo.

##### 3.1.1.5 Selective Adenosine A1 Receptor Agonist (GR79236)

Giffin et al. (2003) conducted a double‐blind, randomised, placebo‐controlled, crossover study where the reflex was elicited at 1.5 times the individual pain threshold using a custom‐built planar concentric electrode. AUC was used for analysis. They administered GR79236 at 10 mg/kg intravenously. They showed a nonsignificant reduction in the ipsilateral (17%) and significant reduction in contralateral (20%) NBR R2 response.

##### 3.1.1.6 Selective Serotonin Receptor Agonist (Oral Zolmitriptan)

Katsarava et al. (2004) used a double‐blind, placebo‐controlled, crossover design where the NBR R2 response was elicited using an intensity 1.5 times the individuals pain threshold and analysed using AUC. They administered zolmitriptan in a 5‐mg dose orally. They found no effect on the NBR in their healthy participants.

##### 3.1.1.7 α2‐Adrenergic Receptor Antagonist (Yohimbine) in a Human Surrogate Pain Model

Vo and Drummond (2016) conducted a double‐blind, placebo‐controlled, crossover study where the NBR R2 response was elicited at 10 times the electrical detection threshold up to a maximum of 8 mA following high‐frequency stimulation (HFS). The NBR R2 response was analysed using AUC. They administered 16 mg of yohimbine orally. They aimed to study whether yohimbine works on the NBR alongside HFS‐induced pain sensitivity. They found an ipsilateral facilitatory effect (approximately 15%) of yohimbine on the NBR. They also found that the contralateral NBR was increased under yohimbine.

#### 3.1.2 Studies Using the Standard Patch Electrode

##### 3.1.2.1 OPs (Fentanyl)

Cruccu et al. (1991) used a single‐blind (participant blinded), placebo‐controlled, crossover where the BR was elicited with stimulation at 50 mA, roughly 10 times the participant's threshold aiming to ensure a painful sensation, with the response measured as a percentage reduction of amplitude. Fentanyl was administered intramuscularly (I.M.) at a dose of 1.5 mg, with naloxone administered as an OP antagonist to reverse the fentanyl effects at a dose of 0.8 mg delivered intravenously (I.V.). They found no effect on the tactile R1 amplitude and a 25% reduction in the nociceptive R2 component in the second of their two testing windows.

##### 3.1.2.2 BZD (Diazepam)

Cruccu et al. (1991) used a single‐blind (participant blinded), placebo‐controlled, crossover design where the BR was elicited with stimulation at 50 mA, roughly 10 times the participant's threshold with the response measured as a percentage reduction of amplitude. They used an I.V. dose of 10 mg for diazepam and tested the effect of a BZD antagonist flumazenil at an I.V. dose of 0.5 mg. They found that diazepam reduced the R2 by 60%, and this was only partially reversed by flumazenil.

##### 3.1.2.3 Nonsteroidal Anti‐Inflammatory Drugs (Piroxicam and ASA)

Fabbri et al. (1992) used a single‐blind, placebo‐controlled, crossover using piroxicam, administered via I.M. injection at a dose of 40 mg where the BR was elicited using an intensity 10 times the sensory threshold (10–80 mA, at a mean of 50 mA) and used AUC for analysis. Ferracuti et al. (1994) used a single‐blind, placebo‐controlled, crossover design testing the effects of ASA and piroxicam in separate sessions; they used an intensity of 10 times the sensory threshold to elicit the reflex (mean of 50 mA) and used AUC for analysis. The R2 response was not significantly different to placebo in either of the studies.

##### 3.1.2.4 Selective Adenosine A1 Receptor Agonist (GR79236)

Giffin et al. (2003) conducted a double‐blind, randomised, placebo‐controlled, crossover study where the reflex was elicited at 1.5 times the individual pain threshold at 12–15 mA using a standard electrode. AUC was used for analysis. They administered GR79236 at 10 mg/kg intravenously. The standard electrode showed no change in R2 response.

##### 3.1.2.5 Nitrous Oxide (NO)

Willer et al. (1985) conducted a double‐blind, randomised, crossover study where the BR was elicited using stimulation intensity 2–3 times the reflex threshold (12–15 mA). AUC was used for analysis. They administered NO (33%) with open‐circuit mask constant flow (101/min). They then attempted reversal with naloxone hydrochloride (1.2 mg; 3‐mL total doses). They found that NO suppressed the R2 response by 83% and decreased pain ratings; however, no reversal was observed. The R1 response was reduced 41%.

##### 3.1.2.6 α2‐Receptor Agonist (Clonidine)

Palmeri et al. (2000) conducted a controlled, within‐subject study where the BR was elicited using stimulation 5 times below the individual detection threshold and always below the pain threshold with single cathodal stimuli. It was analysed using AUC. They administered I.V. 0.5‐μg/kg clonidine (α2 agonist) in 5‐mL saline. They found that the ipsilateral R2 response was reduced 15% versus the control group (but nonsignificant using a significance level of 0.001). They also found that the R1 response was reduced by 43%.

### 3.2 Noradrenergic Modulation of NBR Circuitry

**FIGURE 2:**
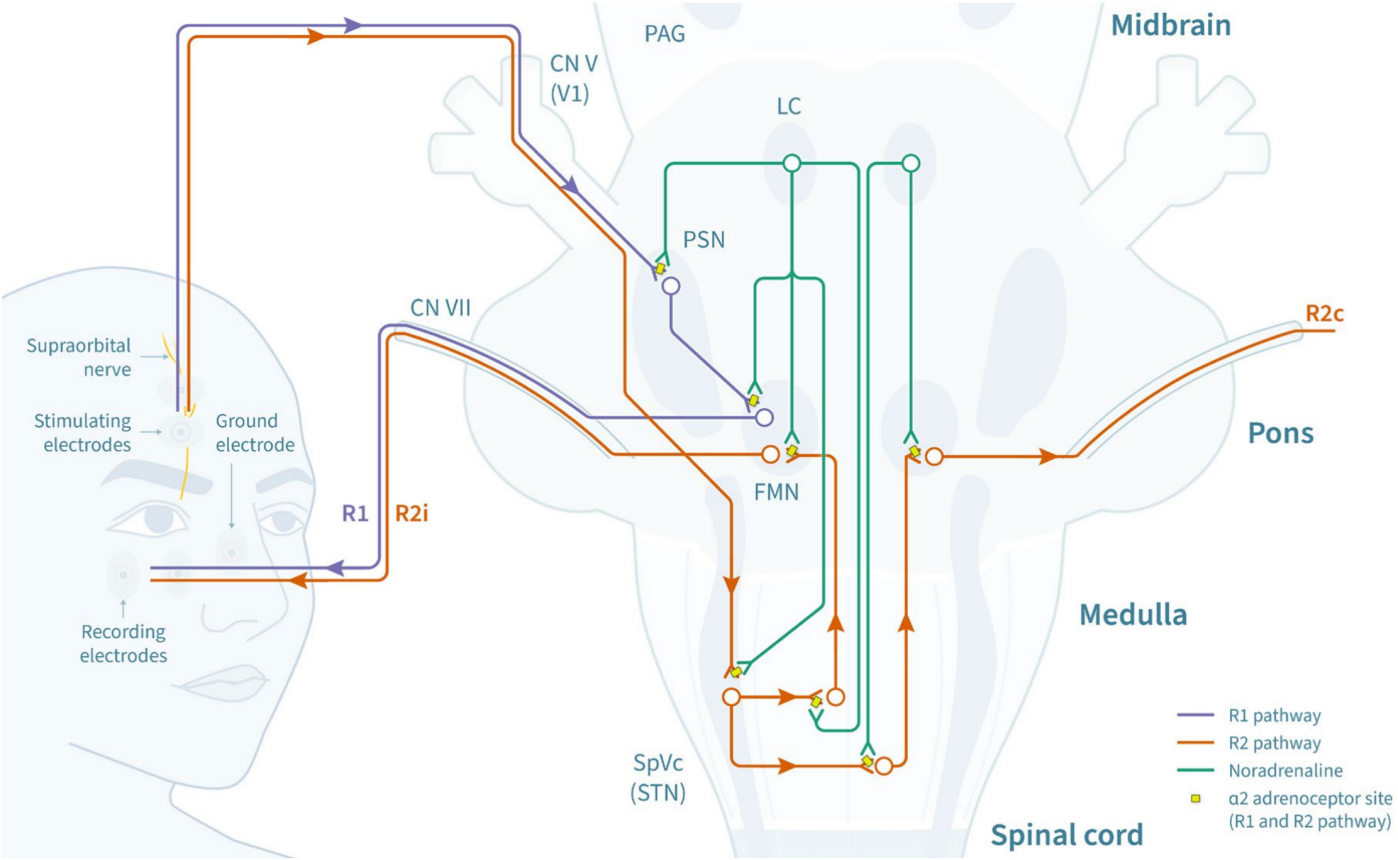
Schematic displaying brainstem pathways. The brainstem taking a cross‐sectional perspective to display the R1 and R2 pathways and how they are modulated by α2‐adrenoreceptor pathways with a primary focus on noradrenergic involvement throughout the brainstem projected from the LC. The R1 pathway is a short‐latency, oligosynaptic, ipsilateral, non‐nociceptive reflex arc. It enters at the level of the pons into the PSN. From there, it travels via a premotor interneuron to the FMN, which drives a response at the orbicularis oculi muscle. The R2 pathway is a longer latency, bilateral, polysynaptic nociceptive reflex. Afferent signals enter at the level of the pons before descending to the STN where a projection interneuron conveys a signal to the lateral tegmental field, where a series of premotor interneurons carry the ipsilateral response to the FMN, which provides the output of the reflex to the orbicularis oculi muscle. For the contralateral R2, an interneuronal axon decussates across the midline before synapsing within the RF, and then with a contralateral premotor interneuron that projects to the contralateral FMN, resulting in a slightly delayed contralateral response. Abbreviations: CN V (V1) = cranial nerve V (V1 entry), CN VII = cranial nerve VII, FMN = facial motor nucleus, LC = locus coeruleus, PAG = periaqueductal grey, PSN = principle sensory nucleus, R2c = contralateral R2 pathway, R2i = ipsilateral R2 pathway, SpVc = spinal trigeminal nucleus caudalis, STN = spinal trigeminal nucleus.

Noradrenergic pharmacological studies most consistently reported modulation of the R2 response using both concentric and patch electrodes. One study found that the synthetic OP fentanyl altered the R2 response (Cruccu et al. 1991), whereas a subsequent study reported no significant effect (Marin et al. 2015). Similarly, no effects of serotonergic pharmacological manipulation on the blink reflex were reported (Katsarava et al. 2004). Administration of the α2‐adrenoceptor agonist clonidine reduced both R1 and R2 responses, whereas the α2‐adrenoceptor antagonist yohimbine facilitated the sensitised NBR. Based on these studies, the proposed endogenous noradrenergic pathway within the R1 and R2 reflex circuits is illustrated in Figure 2.

### 3.3 Risk of Bias Assessment

**FIGURE 3:**
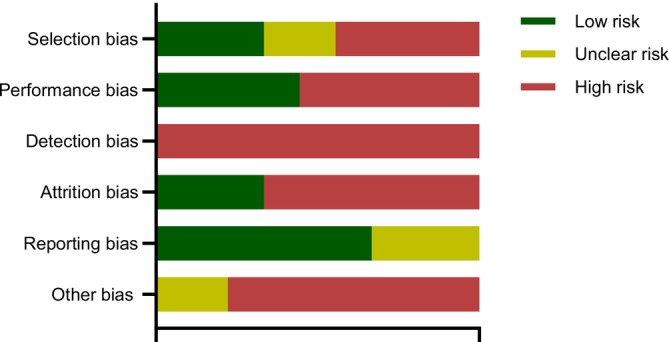
Risk of bias assessment for all included studies. For each study, the risk of bias was assessed based on six standardised domains: selection bias, performance bias, detection bias, attrition bias, reporting bias and other biases. Green indicates the number showing low risk, yellow for an unclear risk and red for high risk. This includes all studies identified (*n* = 9).

Risk of bias was independently assessed by two authors (J.M. and J.K.) using the Cochrane Risk of Bias Tool (Higgins et al. 2011). Six domains were evaluated: selection, performance, detection, attrition, reporting and other bias (Figure 3). Selection bias was assessed according to study design (e.g., randomisation and allocation concealment for interventional studies, placebo group for observational studies). Performance and detection bias were based on the blinding of participants, personnel and outcome assessors, with studies rated as high risk if blinding was not reported. Attrition bias considered incomplete outcome data, reporting bias assessed selective reporting of prespecified outcomes and other bias included factors such as sample size and demographic representation. An in‐depth risk of bias overview is available in the Supporting Information.

## 4 Discussion

In this review, we systematically assessed the literature on the use of pharmacological interventions using the R2 component of the BR in healthy participants evoked using NS concentric and standard patch electrodes. R2 responses evoked using concentric electrodes were sensitive to noradrenergic, but not serotonergic or opioidergic pharmacology. NBR R2 circuitry was also influenced by GABAergic and A1 receptor agonist interventions. Similarly, R2 responses evoked using patch electrodes were sensitive to noradrenergic, but not opioidergic pharmacology. They were also similarly affected by GABAergic interventions, as well as NO. Together, these studies suggest trigeminal nociceptive processing evoked using standard patch and NS concentric electrodes is sensitive to pharmacological manipulation of descending noradrenergic control systems as well as within local excitatory and inhibitory connections within trigeminofacial circuits.

The descending pain modulatory system (DPMS) comprises a complex network of top‐down pathways involving distinct anatomical and pharmacological pathways (Ossipov et al. 2010). These findings suggest that descending serotonergic systems may not exert a significant influence over brainstem R2 circuitry. In contrast, top‐down noradrenergic pathways appear to have a more consistent modulatory role, as evidenced by findings that the α2‐adrenoceptor agonist clonidine inhibits the R1 and the R2 response (Palmeri et al. 2000), whereas the α2‐adrenoceptor antagonist yohimbine facilitates the sensitised R2 response (Vo and Drummond 2016).

Given that clonidine can reduce both the R1 and R2 response (Szabadi 2013), it is possible that noradrenergic modulation occurs via α2 adrenoceptors in the FMN and PSN. The observation that the α2 adrenoceptor antagonist yohimbine facilitates the sensitised R2 response (Vo and Drummond 2016) may also imply increased transmission in the FMN; however, studies indicate that the RF (Aramideh et al. 1997; Gray 2013; Mansouri et al. 2001; Nicholas et al. 1993; Unnerstall et al. 1984) and the STN (Cruccu et al. 2005; Dauvergne et al. 2008; Donertas‐Ayaz and Caudle 2023; May and Warren 2021; Samuels and Szabadi 2008) also serve as relevant sites of action for noradrenaline. The locus coeruleus (LC) is the principal source of noradrenaline to the brainstem and spinal cord and is activated, in part, by the periaqueductal grey (PAG) (Lubejko et al. 2024). Taken together, these lines of evidence suggest that top‐down pontine noradrenergic neurons can inhibit circuitry involved in both innocuous (i.e., R1) and noxious (i.e., R2) BR responses, which is likely due to inhibitory action at presynaptic α2 adrenoceptors in the STN and FMN (Bahari and Meftahi 2019; López‐Córdoba et al. 2022).

GABAergic modulation produced the largest pharmacological effect on the R2 response elicited by both electrode types through BZDs. GABA receptors are located within the FMN and the STN along the R2 pathway (Pellegrini et al. 1995). BZDs potentiate GABA‐A receptor activity and are thought to suppress glutamatergic transmission indirectly by enhancing inhibitory tone (Vinkers and Olivier 2012). GABA‐A receptors are ligand‐gated chloride channels that mediate inhibitory postsynaptic currents on a millisecond timescale, making them well suited to modulate short‐latency reflexes such as the R2 response (Farrant and Nusser 2005). However, GABAergic mechanisms within the brainstem are primarily local, producing short‐loop inhibition (e.g., within the polysynaptic R2 circuit) with limited influence from top‐down descending systems (Kumru et al. 2010). Consequently, although BZDs exert strong effects on the R2, their efficacy in treating chronic pain is poor (Kofler et al. 2024; Wright 2020). Moreover, their clinical utility is restricted by safety concerns and the potential for dependence (Lader 2011).

NO also significantly modulated the R1 and R2 reflex. NO is an inhaled anaesthetic (used at a non‐anaesthetic dose) and analgesic agent known to engage multiple neurotransmitter systems. Unlike BZDs, whose actions are largely confined to local GABAergic inhibition within the brainstem, NO engages both local and descending mechanisms (Emmanouil and Quock 2007; Georgiev et al. 2008), integrating opioidergic, noradrenergic and glutamatergic modulation. NO is typically used as an inhaled sedative and analgesic in dental and surgical procedures or in emergency situations for acute pain (Khinda et al. 2023). It has a rapid onset, but effects are only maintained for a few minutes after the inhalation stops (Jastak and Donaldson 1991). This makes it ideal for acute pain states (i.e., elicitation of BR or dental procedures), however, limits its utility for chronic pain conditions (Chan et al. 2016). Furthermore, although safe in the short term, long‐term effects are less well‐characterised with either prolonged or repeated use, where it carries the risk of haematologic and neurologic risks (Weimann 2003), which argues against its suitability as a viable long‐term pain therapeutic.

Ketamine, a dissociative anaesthetic with potent analgesic properties (O'Brien et al. 2014), exerts its primary effects through noncompetitive antagonism of the *N*‐methyl‐D‐aspartate receptor by reducing postsynaptic excitatory glutamatergic transmission and associated plasticity within sensitised nociceptive pathways (Riccardi et al. 2023). Within the brainstem and R2 circuitry, ketamine is thought to act at multiple levels, including the STN and the RF (Liu et al. 2022; Lydic and Baghdoyan 2002). However, because the R2 reflex when studied in healthy populations is unlikely to involve sensitisation induced within brainstem pathways, it is not surprising that ketamine had no effect on reflex responses (Marin et al. 2015). It is likely that fast AMPA receptor glutamatergic transmission is the primary excitatory receptor mechanism involved in the reflex under normal physiological conditions. This mechanism could present the reason why clinical evidence for the use of ketamine in chronic pain is conflicting (Blonk et al. 2010). Some studies show high‐dose infusions can have effects lasting 4–6 weeks; however, others show no impact vs. placebo (Niesters et al. 2014). The potential side effects and need for constant re‐treatment (Niesters et al. 2014) make ketamine an unsuitable option for chronic pain conditions, especially those that are brainstem‐mediated.

These findings have important clinical implications for understanding how to advance the treatment of pain conditions originating within the brainstem. Because the BR and NBR are purely brainstem‐mediated reflexes, modulation by noradrenergic signalling in healthy participants provides valuable insight into mechanisms that underlie the effects of pharmacological agents in patient populations. Evidence from clinical studies supports the analgesic potential of compounds acting on the noradrenergic system. The serotonin–noradrenaline reuptake inhibitor (SNRI) duloxetine has demonstrated efficacy in both trigeminal neuralgia and persistent idiopathic dentoalveolar pain (Anand et al. 2011). Another SNRI, milnacipran, has shown comparable improvements in chronic orofacial pain (Ito et al. 2010). These findings highlight the therapeutic potential of targeting noradrenergic mechanisms within the brainstem. However, to fully clarify these mechanisms, experimental studies in healthy participants using brainstem reflexes such as the BR are required.

Additionally, the pharmacological sensitivity of the R2 appears to be similar when elicited using standard versus concentric electrodes. Even though these approaches recruit different afferent inputs, with standard electrodes reflecting a convergence of tactile Aβ and nociceptive Aδ fibres onto WDR neurons and concentric electrodes likely activating NS neurons within the STN, their modulation by different classes of pharmacological agents appears consistent. Both showed modulation from noradrenergic and GABAergic influences, whilst showing limited effects of OPs and NSAIDs. Typically, studies using patch electrodes use stimulation intensities in the noxious range (e.g., > 50 mA), which produced a painful response similar to that evoked using NS concentric electrodes. It is therefore possible that modulation of nociceptive responses can occur at both WDR and NS neurons within the subnucleus caudalis region of the STN, which is a key region involved in the processing of trigeminal nociception (Ellrich 2002). The observed noradrenergic pharmacology is therefore likely to reflect modulation of nociceptive processing within these circuits, as well as via downstream facial motor outputs, rather than having a single site of action. Therefore, whilst it is important for a concentric electrode to be used to activate nociceptive afferents, it is likely that comparable R2 circuitry can be activated using standard patch electrodes when delivered using painful stimulation intensities, with the added benefit of also recording the non‐nociceptive R1 component. However, future studies that want to only interrogate purely nociceptive contributions to the R2 component of the reflex should consider using concentric electrodes.

This review has several important limitations that must be considered when interpreting the findings. First, study screening and data extraction were performed by a single reviewer. Although this approach was necessitated by resource constraints, it increases the potential for human error and subjective bias, which may have led to the inadvertent exclusion of relevant studies. Second, a quantitative meta‐analysis was not possible due to incomplete, inconsistent or unclear reporting of outcomes across studies. This restricted us to providing a narrative synthesis, which reduces the strength of our inferences. Consequently, whilst we have summarised the available results, our conclusions remain tentative and should be interpreted with caution rather than as definitive evidence. Third, there was heterogeneity in study design, interventions and outcome measures across the included literature. Furthermore, many studies exhibited high risk of bias, meaning that results should be interpreted with care. Finally, it must be noted that the review primarily focuses on studies using AUC as a measure of the modulation of the reflex. This measure is highly variable and other outcome measures have been explored in both clinical and healthy populations, including habituation (Corrado et al. 2024; Di Clemente et al. 2009), so findings must be taken with caution.

## 5 Conclusion

In conclusion, this review demonstrates that both standard patch electrodes and NS concentric electrodes used to elicit the R2 response provide sensitive and complementary measures for assessing pharmacological modulation of nociceptive responses in the BR pathway. Across studies, noradrenergic mechanisms consistently influenced the R2 component of the blink reflex, whereas serotonergic and opioidergic systems showed little effect when evoked using either electrode type. GABAergic, NO and A1 receptor‐mediated pathways also modulated the R2 reflex, likely through local inhibitory and excitatory connections within trigeminofacial circuits. More research is needed to strengthen our understanding of how endogenous local and top‐down modulatory systems influence nociceptive processing within the BR pathway, which could lead to new mechanism‐driven analgesic strategies for trigeminal and orofacial pain conditions.

## Author Contributions

**Josh Murphy:** conceptualization, methodology, investigation, writing – original draft, writing – review and editing, formal analysis. **Joy Krecke:** writing – review and editing. **Celia Morgan:** writing – review and editing. **Paul H. Strutton:** writing – review and editing. **Kirsty Bannister:** writing – review and editing. **Sam W. Hughes:** conceptualization, formal analysis, investigation, writing – review and editing, writing – original draft, supervision, methodology.

## Conflicts of Interest

The authors declare no conflicts of interest.

## Supporting information




**Table S1:** Per‐study risk of bias. Study by study a risk of bias carried out for all studies according to six categories: selection bias, performance bias, detection bias, attrition bias, reporting bias and other bias. This is explained fully in Section ‘3.1 Risk of bias assessment’. A rating for each paper has been given corresponding to either green—low risk, yellow—unclear risk or red—high risk.

## Data Availability

Data are available upon request.
