# Supplementary material for: Pharmacological Influences on the R2 Blink Reflex Response in Healthy Participants: A Systematic Review
Source: Eur J Neurosci. 2026 Jul 30;64(3):e70646. doi: 10.1111/ejn.70646 (PMC13422242; doi:10.1111/ejn.70646)
Supplement: Supplementary file 1 — Table S1: Per‐study risk of bias. Study by study a risk of bias carried out for all studies according to six categories: selection bias, performance bias, detection bias, attrition bias, reporting bias and other bias. This is explained fully in Section ‘3.1 Risk of bias assessment’. A rating for each paper has been given corresponding to either green—low risk, yellow—unclear risk or red—high risk. [file EJN-64-0-s001.docx]

| **Study** | **Selection bias** | **Performance bias** | **Detection bias** | **Attrition bias** | **Reporting bias** | **Other bias** |
| --- | --- | --- | --- | --- | --- | --- |
|  |  |  |  |  |  |  |
| Vo and Drummond (2016) |  |  |  |  |  |  |
| Marin et al. (2015) |  |  |  |  |  |  |
| Katsarava et al. (2004) |  |  |  |  |  |  |
| Giffin et al. (2003) |  |  |  |  |  |  |
| Palmeri et al. (2000) |  |  |  |  |  |  |
| Ferracuti et al. (1994) |  |  |  |  |  |  |
| Fabbri et al. (1992) |  |  |  |  |  |  |
| Cruccu et al. (1991) |  |  |  |  |  |  |
| Willer et al. (1985) |  |  |  |  |  |  |
|  |  |  |  |  |  |  |
| Key: |  |  |  |  |  |  |
| **Low** |  |  |  |  |  |  |
| **Unclear** |  |  |  |  |  |  |
| **High** |  |  |  |  |  |  |

**Supplementary table 1. Per-study risk of bias.** Study by study a risk of bias carried out for all studies according to 6 categories: selection bias, performance bias, detection bias, attrition bias, reporting bias and other bias. This is explained fully in section ‘3.1 Risk of bias assessment’. A rating for each paper has been given corresponding to either green – low risk, yellow – unclear risk or red – high risk.

Our risk of bias assessment revealed mixed findings across domains. Approximately one-third of the studies fell into each category of selection bias (low, unclear, or high risk), primarily due to insufficient reporting of participant selection and allocation procedures. Performance bias was identified as high risk in over 50% of studies, largely attributable to a lack of detail regarding blinding of participants and personnel or control conditions. Detection bias emerged as the most concerning domain, with all studies rated as high risk due to the absence of blinding during outcome assessment. In contrast, reporting bias was minimal; all studies clearly stated their objectives, and none were rated as high risk. For attrition bias, most studies were rated as high risk due to incomplete or unclear reporting of participant dropouts and their reasons. Other sources of bias were also frequently rated as high risk, commonly due to small sample sizes or imbalanced sex distribution, both of which may limit the generalisability of findings.
